# Traumatic Pericarditis in a Cow

**Published:** 1903-04

**Authors:** John J. Repp

**Affiliations:** Ames, Iowa


### REPORTS OF CASES.

### TRAUMATIC PERICARDITIS IN A COW.

By John J. Repp, V.M.D.,
AMES, IOWA

April 1, 1901, I was asked to see a three-year-old pure-bred short-horn cow which the herdsman told me had been sick for about a month, lately growing rapidly worse. I found marked œdema about the dependent parts of the body, well-developed serous infiltration of the pleural and the peritoneal cavity, temperature 103°, pulse rapid and small, respiration rapid, and expiration grunting. I made a diagnosis of traumatic pericarditis, gave a prognosis of fatal termination, and advised destruction. The man in charge, however, said he was anxious to try some treatment, as the cow was valuable. Accordingly I prescribed a powder of digitalis, calomel, and squill, to be given with her feed, advised rest and moderate diet. The cow lived until April 11th, on which date I determined to use her as a subject for the demonstration of *paracentesis thoracis et abdominis* to the senior students of the Veterinary Department, Iowa State College. I was not careful as to the amount of fluid withdrawn, as the operation was solely for demonstrative and not for therapeutic purposes. I drew off from the thorax about a gallon of blood-tinged serum, and a like amount from the abdomen. Within an hour after the operation the cow died, death having been hastened, perhaps, by the sudden withdrawal of a considerable amount of fluid.

Autopsy was made at once. The pleural and the peritoneal cavity each contained a large quantity of clear, sanguinolent fluid. The pericardial sac was distended so as to measure about a foot through each axis, and contained about a gallon of yellow, flaky, puriform fluid of bad odor. The pericardium and its contents, including the heart, weighed twenty-five and one-half pounds. The parietal pericardium was one-eighth to one-fourth inch thick as measured in different places, and its inner face and the surface of the epicardium were roughened and covered with a yellow, spongy, fibrinous exudate one-fourth to one-half inch thick, slightly adherent and removable in large cakes. The myocardium and endocardium appeared to the naked eye to be free from any lesion. The lower part of the lung was carnified by the long-continued pressure of the enormously distended pericardial sac. The reticulum contained about a dozen tacks and nails, several of which had penetrated toward the diaphragm. Leading from the reticulum through the diaphragm to the pericardium was a large fibrous mass burrowed in an irregular, indefinite manner by several tracts containing pus. The offending object was not found, although a careful search was made for it in the fibrous mass and in the sac. There is a probability that it passed back again into the reticulum after inciting the inflammatory reaction upon its forward passage.

